# Clinical management and therapeutic development for the rare disease rhabdomyosarcoma

**DOI:** 10.7150/jca.127496

**Published:** 2026-01-01

**Authors:** Ting-Ling Ke, Linyi Chen

**Affiliations:** 1 Institute of Molecular Medicine, National Tsing Hua University, Hsinchu, Taiwan, No. 101, Section 2, Kuang-Fu Road, Hsinchu, 30013, Taiwan.; 2 Department of Medical Science, National Tsing Hua University, Hsinchu, Taiwan, No. 101, Section 2, Kuang-Fu Road, Hsinchu, 30013, Taiwan.

**Keywords:** rhabdomyosarcoma, myogenesis, myoblast fusion, targeted therapy, rare disease

## Abstract

Rhabdomyosarcoma (RMS) is a rare disease that arises from skeletal muscle mainly affects children and adolescents. Patients with RMS have diverse symptoms and prognosis based on tumor sizes, tumor anatomical locations, histological subtypes of the tumors and genetic testing of *paired-box-forkhead box O1* (*PAX-FOXO1*) fusion gene. The 4 subtypes of RMS include embryonal RMS (eRMS), alveolar RMS (aRMS), spindle cell/sclerosing RMS (scRMS) and pleomorphic RMS (pRMS). Treatment for RMS patients remains challenging due to its heterogeneous nature. Thus, a combinatory approach is likely to warrant better management of RMS. Given that *PAX-FOXO1* fusion gene is the most common biomarker for RMS, though this fusion gene only accounts for 16-20% of RMS patients. Targeted therapy that tailors treatment plans to the individual patient may provide additional benefits for RMS patients. This review describes the frequent genetic mutations observed in RMS patients and drug development based on these mutations shall provide direction to develop targeted therapy leading to effective personalized treatment for RMS patients.

## 1. Skeletal muscle diseases and related rare diseases

Skeletal muscles are essential for movement, breathing, posture, and overall health. Disorders affecting the skeletal muscle tissue are categorized as musculoskeletal and neuromuscular disorders. Musculoskeletal disorders involve injuries and conditions affecting the skeletal muscles and associated connective tissues such as bones and joints. The symptoms of musculoskeletal disorders include pain, stiffness, limited range of motion, inflammation, and fatigue. Musculoskeletal disorders could also contribute to muscle atrophy including muscular dystrophy, osteoarthritis, rheumatoid arthritis, and spinal muscular atrophy. Neuromuscular disorders affect the peripheral nerves that control voluntary muscles or neuromuscular junction. Muscular dystrophy results in wasting and loss of muscle tissue, disability and possible deformity. Treatment for muscle atrophy includes regular exercise, physical therapy, medications managing chronic diseases or addressing nutritional deficiencies. Uncontrolled cell growth arising from bones or muscles will lead to musculoskeletal cancers, which are often rare cancers, such as osteosarcoma, Ewing sarcoma, chondrosarcoma, rhabdomyosarcoma (RMS), leiomyosarcoma. The treatment for musculoskeletal cancer depends on the sizes, stages and locations of the cancers which usually involves surgery, radiotherapy and chemotherapy. Targeted therapy provides more personalized and effective treatment approaches and play an important role in the management of some cancers such as breast cancer, lung cancer and colorectal cancer. Although there is some targeted therapy for osteosarcoma 1, 2, much less is available for musculoskeletal cancer compared to other cancer types.

## 2. Histological subtypes of RMS

RMS is a rare disease, as defined by Genetic and Rare Diseases Information Center (GARD), National Institute of Health (NIH). RMS is the most common type of soft tissue sarcoma that malignant cells arising from skeletal muscle, and it primarily affects children and adolescents. Approximately 350 new cases of RMS are diagnosed every year in the United States. Overall incidence is 4.5-6 cases per million people in Europe and United States and 3.5 cases per million in Asia 3. Although the overall five-year survival rate for RMS in children exceeds 70%, the five-year survival rate of children with RMS that has metastasized to distant parts of the body is less than 30% and patients with recurrent RMS is only 17% 4-6. Adults with RMS have worse survival than children, with an overall five-year survival rate of only 20% to 40% 7, 8.

**Figure 1:**
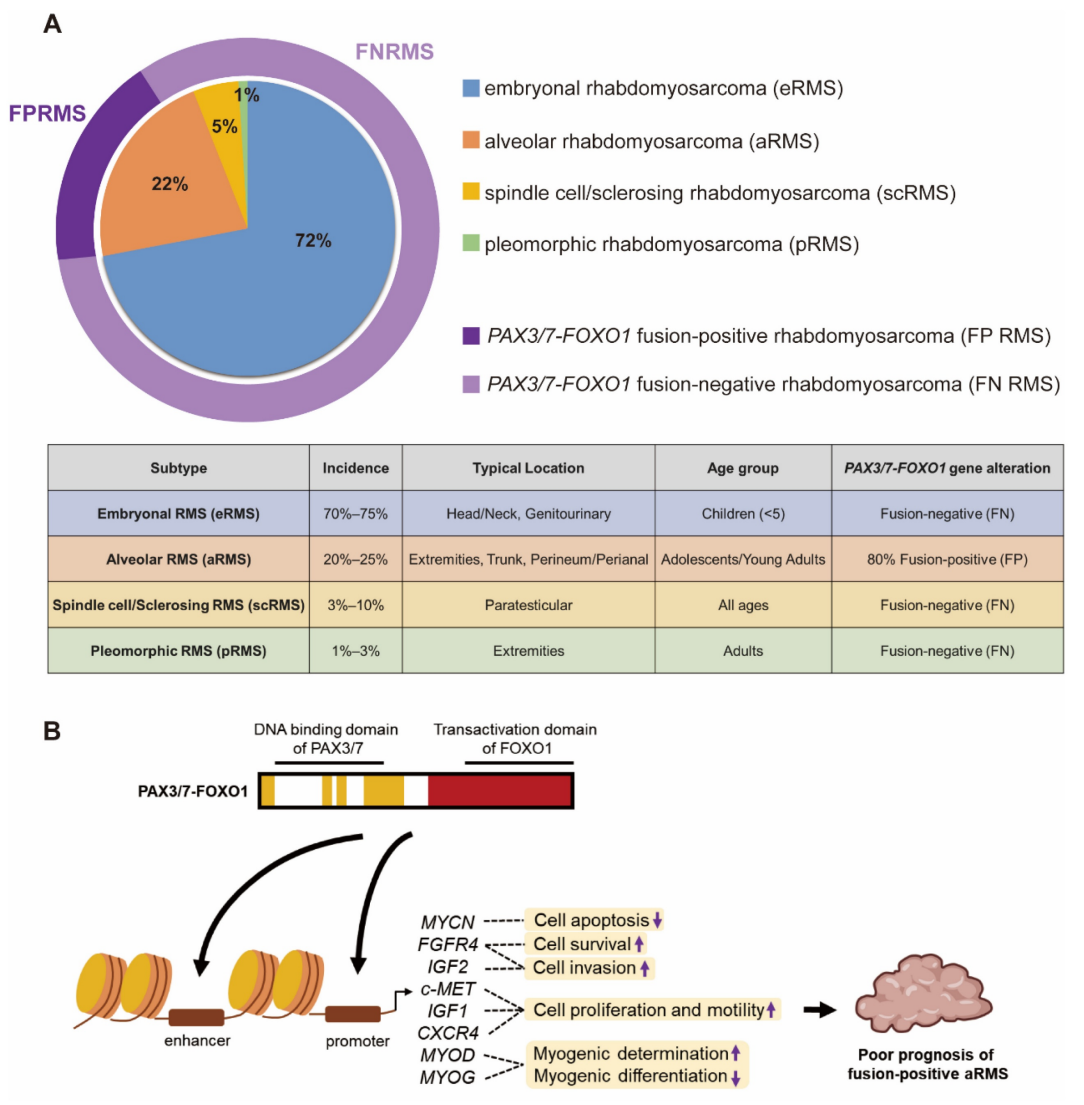
** Subtypes of RMS and PAX3/7-FOXO1 fusion protein in FP aRMS.** (A) The incidence, tumor location, age group and *PAX3/7-FOXO1* fusion gene alteration of different RMS subtypes. (B) The PAX3/7-FOXO1 fusion protein drives epigenetic changes and alters genes transcription, leading to poor prognosis of FP aRMS.

There are 4 histologic subtypes of RMS classified according to the 5th edition of the World Health Organization (WHO) Classification of Tumors of Soft Tissue and Bone: embryonal rhabdomyosarcoma (eRMS), alveolar rhabdomyosarcoma (aRMS), spindle cell/sclerosing rhabdomyosarcoma (scRMS) and pleomorphic rhabdomyosarcoma (pRMS) (Figure 1A) 9. The embryonal subtype is the most frequently observed subtype which account for 70% to 75% of childhood RMS. eRMS is associated with round-cell phenotype and typically arises in the head and neck or genitourinary region in children younger than five. Approximately 20% to 25% of children with RMS have the alveolar subtype. Tumors with alveolar histology usually in extremities, trunk, and perineum/perianal region and have an increased frequency in adolescents and young adults 10. The scRMS are considered in the same diagnosis spectrum and account for 3% to 10% of all cases, with high frequency at the paratesticular site. The pRMS is an extremely rare subtype characterized by a high-grade pleomorphic sarcoma arises in the extremities in adults 9. This type of RMS often results in poor prognosis. Additionally, RMS has been further characterized based on molecular biology characteristics of fusion proteins paired-box 3-forkhead box O1 (PAX3-FOXO1) or paired-box 7-FOXO1 (PAX7-FOXO1). Here, fusion-positive (FP) and fusion-negative (FN) were defined 6, 11. Approximately 80% of patients with the aRMS (16% of all RMS) have chromosomal translocations resulting in *FOXO1* gene fusion to *PAX3* or *PAX7*, which is implicated in a poorer prognosis 12, 13. Fusion between DNA binding domain of PAX3/7 and transactivation domain of FOXO1 drives epigenetic changes and alters hundreds of genes transcription. Several studies have implicated that PAX3/7-FOXO1 fusion protein regulates many downstream factors to suppress cell apoptosis (MYCN) 14, promote cell survival (FGFR4), enhance cell invasion (FGFR4 and IGF2) 15, 16, increase cell proliferation and motility (c-MET, IGF1, CXCR4) 17, 18, drive myogenic determination and repress myogenic differentiation (MYOD, MYOG) 14, 19, 20 (Figure 1B). These *PAX3/7-FOXO1*-mediated genomic instability is strongly associated with a poor prognosis in FP aRMS 20.

## 3. Clinical treatments of RMS

Treatment of RMS presents unique challenges due to the scarcity of the disease and various anatomical sites of primary tumor. For optimal management and treatment, patients with RMS require multimodality therapy including surgery, radiation therapy and systemic chemotherapy to ensure receiving ideal treatment, supportive care and rehabilitation to achieve optimal survival and quality of life. 21-23. Optimizing patient care requires tailored treatment decisions regarding surgical and radiotherapeutic options, which must be based on factors such as patient ages, tumor sizes, histological subtypes and the anatomical locations of tumors. If the tumor resection will not cause dysfunction or deformity, surgical resection is performed first before other treatment. If this is not possible, only an initial biopsy is performed. Radiation therapy is often used for RMS patients with metastasis or the tumors that cannot be completely removed surgically. In order to prevent recurrence or metastasis of RMS, chemotherapy is used after surgery and/or radiation therapy. The standard chemotherapy regimen for patients with RMS is the combination of vincristine, actinomycin D, and cyclophosphamide (VAC) in North America 24. In Europe, the chemotherapy regimen is the combination of ifosfamide, vincristine, and actinomycin D (IVA) 6. Vincristine binds irreversibly to microtubules and spindle proteins in S phase of the cell cycle and thus interferes with the formation of the mitotic spindle, thereby arresting tumor cells in metaphase. Actinomycin D binds to guanine residues in DNA and blocking the action of DNA-dependent RNA polymerase functions thereby inhibit RNA synthesis. Cyclophosphamide and ifosfamide react with DNA to interfere DNA replication and cell division. There are no significant differences in the clinical outcomes between VAC and IVA regimens 25. Table 1 listed the ongoing or completed clinical trials related to RMS. Most clinical trials focus on the combination of common chemotherapy drugs, while only limited trials test antibody or T cell transplantation application in RMS.

## 4. Genetic mutations in patients with RMS

**Figure 2:**
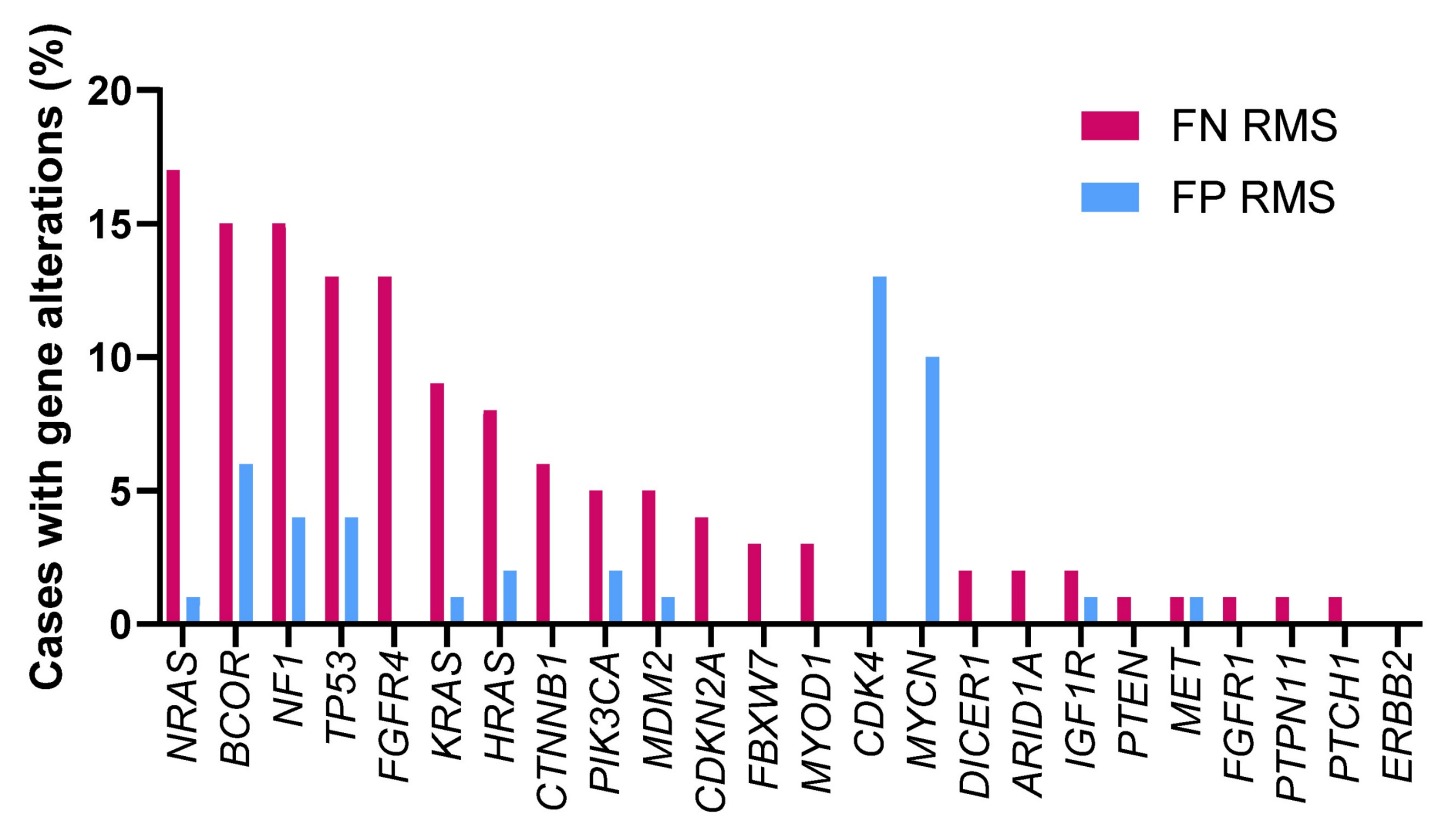
** Gene alterations in FN RMS and FP RMS.** The percentage of cases with gene alterations in FN and FP RMS patients were shown. The data used to generate this summary graph were based on results from J.F. Shern *et al.* 30.

Previous reports have documented the mutations observed in eRMS (28.3%) and aRMS (3.5%) 26. A wide range of genetic mutations were identified in FN RMS including mutations in *NRAS*, *KRAS*, *HRAS* 27, *TP53* 28, *PIK3CA*, *CTNNB1* 26 and *FGFR4* 29. These gene mutations in RMS are associated with more aggressive genotypes and poorer outcomes. Multiple mutations within individual tumors in FN RMS are associated with worse event-free survival 30. Thus, it is critical to further characterize the genetic events underlying RMS in order to develop more effective diagnostic, prognostic and therapeutic strategies. J.F. Shern *et al.* compared gene mutations between FN and FP RMS patients using a combined cohort of the Children's Oncology Group (COG) and United Kingdom RMS patients (n = 641) 30 (Figure 2). A higher frequency of mutations in *NRAS*, *BCOR* and *NF1* genes was observed in patients with FN RMS while mutations in *CDK4* and *MYCN* are more common in patients with FP RMS. The differential frequency of gene mutations between FN and FP RMS may provide direction in developing personalized medical approaches.

### 4.1 *NRAS* and *NF1* mutations

The *RAS* gene encodes a membrane-bound GTPase which cycles between an active GTP-bound state and an inactive GDP-bound state. Three members of RAS family NRAS, (Neuroblastoma Rat Sarcoma Virus), HRAS (Harvey Rat Sarcoma Virus) and KRAS (Kirsten Rat sarcoma virus), that transmit signal transduction to regulate a variety of cellular process such as cell growth, differentiation, survival and apoptosis 31-34. After receptor tyrosine kinase (RTK) engagement, guanine nucleotide exchange factors (GEFs) such as SOS1, SOS2 and RASGRF are recruited to the plasma membrane to regulate RAS activity through the exchange of GDP for GTP on RAS proteins 35. Conversely, RAS switches to the inactive form catalyzed by GTPase-activating proteins (GAPs), which in turn accelerates the hydrolysis of bound GTP to GDP 36, 37. There are two major downstream signaling pathways of RAS including RAF/MEK/ERK (MAPK) and phosphatidylinositol-3-kinase (PI3K)/protein kinase B (AKT) cascades 38. The RAS/RAF/MEK/ERK pathway plays a crucial role in cell proliferation, migration and invasion of cancer cells 39, 40. Active RAS recruits RAF kinase to the plasma membrane, where RAF is activated. Active RAF then phosphorylates and activates MEK which subsequently activate ERK. Active ERK can translocate to the nucleus and activate transcription factors to regulate cell cycle progression 40, 41. PI3K/AKT/mammalian target of rapamycin (mTOR) signaling cascade is also the well-studied downstream pathway of RAS which regulates cell progression, protein synthesis, metabolism and cell survival 42. RAS activates PI3K signaling which phosphorylates phosphoinositides and generates phosphatidylinositol 3,4,5-triphosphate (PIP3), which activates AKT and its substrates, including mTOR, FOXO or NF-κB. The lipid phosphatase PTEN can negatively regulate the PI3K/AKT pathway by dephosphorylating PIP3 and thus reduces the level of phosphorylated AKT. More than 20 percent of cancers contain mutations of RAS family proteins 43, and this accounts for 35% in RMS 27, 44, 45. Additionally, hypermethylation of the *PTEN* promoter is found in 90% of FN RMS tumors 46. Decreased expression level of PTEN or the mutation of *PTEN* also present in a subset of FN RMS 30, 47, 48. *KRAS* mutations are found in approximately 22% of cancers, especially in adenocarcinoma. *NRAS* mutations present in 8% of tumors with high frequency in melanoma. Only 3% of tumors have *HRAS* mutations 49, 50. The RAS mutant defective in GTP hydrolysis can lead to accumulation of GTP-bound RAS and constitutive RAS activation 51 and increase the affinity of NRAS to RAF-1 52 and PI3K 53.

*NF1* encodes neurofibromin 1 containing a region which is similar to the catalytic domain of GTPase-activating proteins (GAPs) 54. NF1 stimulates GTPase activity of RAS protein, thereby promoting the formation of inactive RAS-GDP and turning off downstream signaling. Tumors associated with *NF1* mutations include glomus tumor 55, optic glioma 56, juvenile xanthogranulomas 57, gastrointestinal stromal tumor 58 and RMS 30. Inactivating mutations of NF1 results in accumulation of active RAS-GTP leading to constitutive activation of the RAS signaling pathway. Increased active RAS-GTP levels stimulate RAS/RAF/MAPK signaling pathway and PI3K/AKT/mTOR signaling pathway which ultimately cause increased cell proliferation and inhibit cell apoptosis.

**Figure 3:**
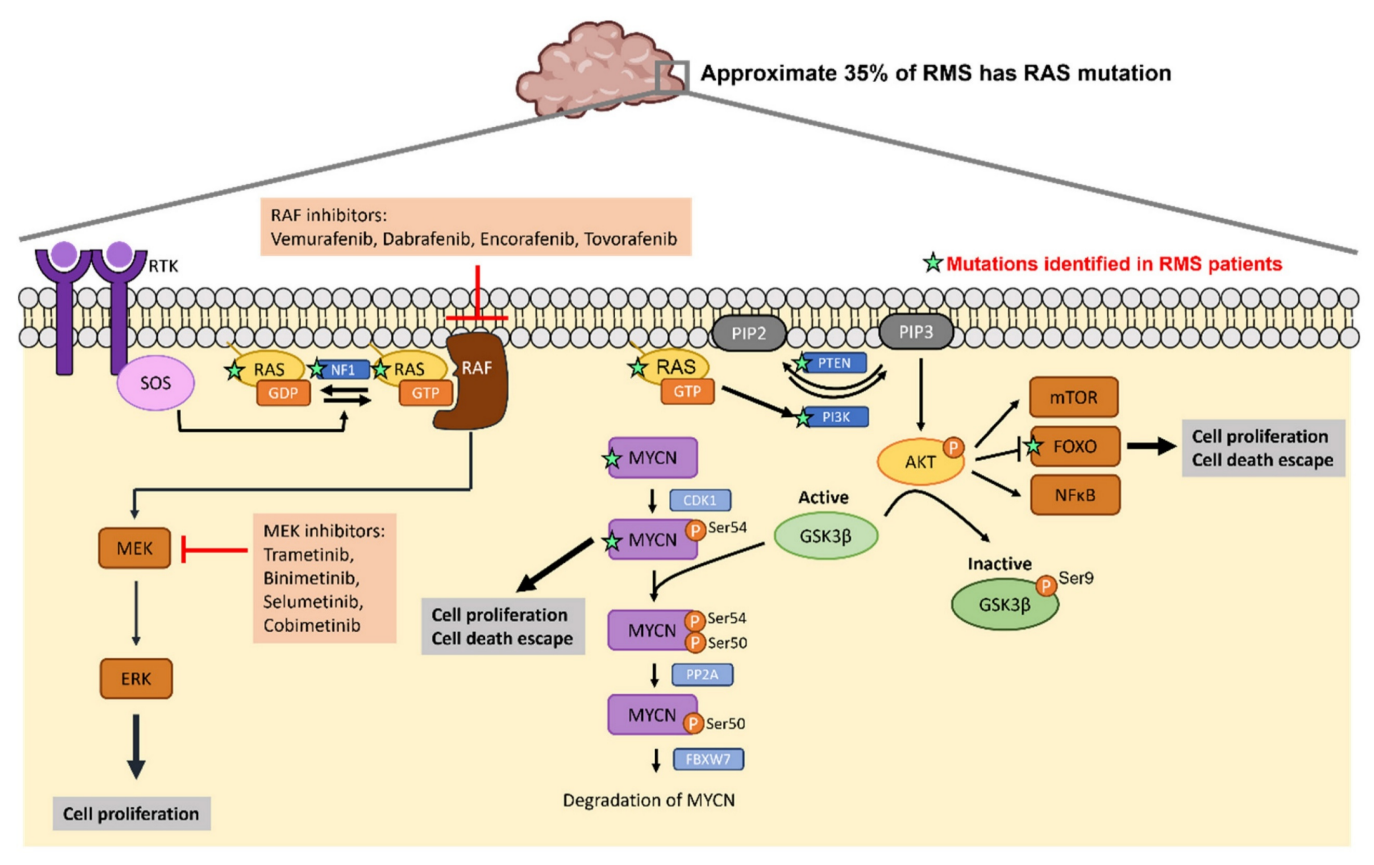
** RAS mutations in RMS and the corresponding drugs that target RAS signaling cascades.** Activation of RTK leads to RAS switch to the active RAS-GTP. RAS-GTP activates RAF/MEK/ERK and PI3K/AKT pathways leading to increased cell proliferation and cell death escape. FDA approved RAF inhibitors and MEK inhibitors can inhibit RAS signaling cascades. The green stars label the mutations identified in RMS patients.

Given the high recurrence in cancers such as colorectal cancer, pancreatic cancer and lung cancer, signaling molecules within RAS pathways are potential targets to develop targeted therapeutic strategies. Previous studies have reported small molecules that bind to RAS directly to affect its GTP-GDP regulation 59, 60 or to inhibit the interaction between RAS and GEFs 61, 62. Table 2 lists the ongoing or completed clinical trials that target downstream effectors of RAS pathway in cancers with RAS mutations. Figure 3 summarizes the mutated genes of FN RMS in RAS pathway and the FDA approved drugs applied to target RAS signaling cascades. The USA FDA has approved four MEK inhibitors for cancer treatment including trametinib, binimetinib, selumetinib and cobimetinib. Several RAF inhibitors have been approved by the USA FDA for treating certain cancers including vemurafenib, dabrafenib, encorafenib and tovorafenib. The confirmed tumor response rate of *BRAF* mutation-positive, unresectable or metastatic melanoma patients is higher with the treatment of trametinib (22%) compared to chemotherapy (8%). The overall response rate of *BRAF* mutation-positive, unresectable or metastatic melanoma patients is higher with the treatment of binimetinib plus encorafenib (63%) compared to vemurafenib (40%). The overall response rate of *BRAF* mutation-positive metastatic non-small cell lung cancer patients is higher with the treatment of dabrafenib plus trametinib (61%) compared to dabrafenib monotherapy (27%). Drugs targeting RAS pathway have been applied to patients with *BRAF* mutation-positive melanoma, glioma or on-small cell lung cancer. Specifically, *RAS* mutations have been found in approximate 35% of RMS patients especially in FN RMS 27, 44, 45. Thus, targeting RAS pathway may have potential for treating RMS.

### 4.2 *CDK4* mutations

Cyclin-dependent kinases (CDKs) are a family of serine/threonine kinases whose activity depends on a regulatory subunit cyclin and play a crucial role in regulating the cell cycle, apoptosis 63, gene expression 64, and cell differentiation 65. CDK4 conjugates with cyclin D to drive the transition from G1 to S phase during DNA replication. The CDK4-cyclin D complex and phosphorylates the retinoblastoma (RB) protein which then releases the E2F transcription factor to initiate gene expression for DNA synthesis. CDK-interacting protein/kinase inhibitory protein (CIP/KIP) family and inhibitors of CDK4 (INK4) family are cyclin-dependent kinase inhibitors. CIP/KIP family members can either inhibit or promote the activity of all major cell cycle CDK/cyclin complexes depending on their posttranslational modification 66. INK4 family members are able to bind only to CDK4/6-cyclin D complexes and block the progression of the cell cycle 67. Deregulation of the CDK4/6-cyclin D-INK-RB pathway has been found in a variety of cancers. Hyperactivated CDK4/6 has been reported in many human cancers as a result of overexpression of cyclin D, inactivation of INK4 and CIP/KIP inhibitors or deletion and/or epigenetic alterations of RB 68. The FDA approved drugs targeting CDK4/6 are listed in Table 3. CDK4/6 inhibitors are commonly used to treat subtypes of breast cancer. Only abemaciclib is used as monotherapy while other drugs are generally used in combinatory therapy. In addition to breast cancer, there are some ongoing and completed clinical trials applying CDK4/6 inhibitors to other cancers (Table 4).

### 4.3 *BCOR* mutations

The BCL6 corepressor (BCOR) was identified as a corepressor interacting with the POZ domain of BCL6. BCOR is a transcription factor which is involved in hematopoiesis, lymphoid development, pluripotency of embryonic stem cells and osteogenic/dentinogenic capacity of mesenchymal stem cells 69, 70. BCOR can form polycomb repressive complex 1 (PRC1) with RING1, RYBP, NSPC1 and associates with the lysine demethylase 2B (KDM2B) protein to mediate transcriptional repression through epigenetic modifications of histones 71, 72. It is a tumor suppressor gene and its loss-of-function mutation has been reported in both malignant and nonmalignant hematologic diseases and is frequently found in acute myeloid leukemia (AML) patients 73-75. Mutations of *BCOR* interrupt assembly of a non-canonical PRC1.1 complex by unlinking the enzymatic core from the chromatin-targeting complex. As a result, BCOR-mutated PRC1.1 can localize to chromatin without repressive activity, resulting in epigenetic reprogramming and aberrant transcriptional activation of oncogenic signaling programs 72. Patients with *BCOR* mutation have a reduced survival rate and poor prognosis since *BCOR* mutations have been linked to resistance to certain chemotherapy drugs 74, 76. While there aren't currently any drugs specifically targeting *BCOR* mutations, researchers are exploring targeted therapies based on the mechanisms through which *BCOR* mutations drive cancer development. Approximately 65% of uterine sarcoma patients with *BCOR*-rearranged mutations have *CDK4* amplification or *CDKN2A* gene inactivation and multiple genes in the CDK4 pathway are overexpressed in *BCOR-CCNB3*-fused sarcomas 77, 78, suggesting potential targeted therapeutic implications of CDK4/6 inhibitors in *BCOR* mutated patients.

### 4.4 *MYCN* mutations

The *MYCN* gene is a member of the *MYC* oncogene family, which consists of *MYCC*, *MYCN* and *MYCL*. The MYC family plays a crucial role in governing the gene expression related to cell proliferation, differentiation, protein synthesis, metabolism and apoptosis 79-81. *MYCN* gene encodes a transcription factor N-MYC with a short half-life around 30 min 82 whose stability is related to phosphorylation on specific residues. First, CDK1-cyclin B1 phosphorylates N-MYC at Ser54. Then serine-threonine kinase glycogen synthase kinase 3β (GSK3β) recognizes N-MYC (pSer54) and phosphorylates it at Thr50 subsequently triggering protein phosphatase 2A (PP2A)-dependent dephosphorylation of Ser54. Next, F-box and WD repeat domain-containing 7 (FBXW7) polyubiquitinates N-MYC leading to proteasomal degradation 83-85. When the PI3K/AKT pathway is activated, active phospho-AKT phosphorylates and inactivates GSK3β resulting in stabilization of N-MYC 86, 87. The deregulation of *MYCN* occurs in many kinds of cancers and is related to poor prognosis. Amplification of *MYCN* has been observed in 17-22% of all neuroblastomas 88, 89 and 5-50% of medulloblastomas 90-92. Approximately 25% of aRMS cases have amplification of *MYCN* and 55% of aRMS cases have overexpression of *MYCN* 93, 94. Previous studies also showed amplification or overexpression of *MYCN* in neuroendocrine prostate cancers, prostate adenocarcinomas, small-cell lung cancers and breast cancer 95-98. These findings of aberrant expression of *MYCN* in a variety of cancers suggest N-MYC as a therapeutic target.

## 5. Conclusion and future perspectives

Cancer biomarkers are useful for cancer diagnosis, prognosis, and treatment response. Biomarker testing is an important part of precision medicine such as targeted therapy to tailor treatment plans to an individual patient. The field of targeted therapy in cancer management is rapidly evolving because of new discovery of molecular targets, development of new targeted drugs and exploration of different combination strategies.

RMS is a rare cancer that can start in any soft tissues. The diagnosis is performed by conducting physical exams and scans such as MRI, CT or a bone scan. The first line therapy of RMS involves conventional chemotherapy and radiotherapy that affects all rapidly dividing cells. Developing targeted therapies and immunotherapies would be more specific to RMS and lead to less side effects. Once RMS being diagnosed, genetic testing of *PAX-FOXO1* fusion gene would be conducted. The constitutively nuclear-localized PAX-FOXO1 fusion protein drives tumorigenesis and is associated with poor prognosis in FP aRMS by modulating the transcription of several genes such as *FGFR4*, *IGF2* and C*XCR4*. While *PAX-FOXO1* fusion gene was observed in 16% of all RMS cases, identifying other gene mutations with a high frequency of occurrence would provide a more comprehensive and efficient therapeutic approach. This review summarizes the potential biomarkers in RMS. A high frequency (15%) of mutations in *NRAS*, *BCOR* and *NF1* genes were observed in FN RMS while mutations in *CDK4* and *MYCN* are more common in FP RMS. There are several developing drugs and clinical trials targeting to these mutations. If the specific genetic mutation can be detected during early diagnosis, targeted therapy would provide more appropriate and effective personalized treatment for RMS patients.

In addition to targeted therapy, studies also reveal the potential application of immunotherapy in RMS. Specific antigens of RMS were identified as immune markers 99-101. Lavoie RR *et al.* reported the novel mechanistic insights on the role of B7-H3 in tumor immune evasion and RMS progression 99. Cell-based immunotherapy using EGFR-CAR NK cell confers high efficiency against chemotherapy-resistant RMS cells 102. Nonetheless, cancers treated with a single-agent therapy would eventually acquire resistance leading to reduced sensitivity during subsequent treatment lines. The combination of targeted therapy and immunotherapy in RMS may overcome drug resistance and improve the outcomes for patients while reducing the side-effects and providing a long-lasting defense against relapses.

## Figures and Tables

**Table 1:** Ongoing or completed clinical trials related to RMS

NCT number (Registration Date)	Title	Phase#	Status	Study completion date
NCT01355445 (2011-05-16)	Vincristine and Irinotecan with or Without Temozolomide in Children and Adults with Refractory/​Relapsed Rhabdomyosarcoma (VIT-0910)	2	Completed	2019-05
NCT03041701 (2017-02-02)	Insulin-like Growth Factor 1 Receptor (IGF-1R) Antibody AMG479 (Ganitumab) in Combination with the Src Family Kinase (SFK) Inhibitor Dasatinib in People with Embryonal and Alveolar Rhabdomyosarcoma	2	Terminated	2021-10-16
NCT02239861 (2014-09-11)	TAA-Specific CTLS for Solid Tumors (TACTASOM)	1	Completed	2022-04-07
NCT00132158 (2005-08-17)	ZD1839 and Oral Irinotecan in Treating Young Patients with Refractory Solid Tumors	1	Completed	2011-10
NCT02509598 (2015-07-24)	A Study of Lymphoseek® as a Lymphoid Tissue Targeting Agent in Pediatric Patients with Melanoma, Rhabdomyosarcoma, or Other Solid Tumors Who Are Undergoing Lymph Node Mapping	2	Completed	2019-03-06
NCT00075582 (2004-01-09)	Vincristine, Dactinomycin, and Cyclophosphamide with or Without Radiation Therapy in Treating Patients with Newly Diagnosed Low-Risk Rhabdomyosarcoma	3	Completed	2021-09-30
NCT01222715 (2010-10-15)	Vinorelbine Tartrate and Cyclophosphamide in Combination with Bevacizumab or Temsirolimus in Treating Patients with Recurrent or Refractory Rhabdomyosarcoma	2	Completed	2015-06
NCT00025363 (2001-10-11)	Comparison of Chemotherapy Regimens in Treating Children with Relapsed or Progressive Rhabdomyosarcoma	2	Completed	2007-10 (primary completion)
NCT02372006 (2015-02-24)	Trial of Afatinib in Pediatric Tumours	1, 2	Completed	2020-08-05
NCT00001566 (1999-11-03)	A Pilot Study of Autologous T-Cell Transplantation with Vaccine Driven Expansion of Anti-Tumor Effectors After Cytoreductive Therapy in Metastatic Pediatric Sarcomas	2	Completed	2008-09
NCT00001564 (1999-11-03)	A Pilot Study of Tumor-Specific Peptide Vaccination and IL-2 With or Without Autologous T Cell Transplantation in Recurrent Pediatric Sarcomas	2	Completed	2007-10-25
NCT03441360 (2018-02-15)	Study to Assess Safety and Preliminary Activity of Eribulin Mesylate in Pediatric Participants with Relapsed/​Refractory Rhabdomyosarcoma (RMS), Non-rhabdomyosarcoma Soft Tissue Sarcoma (NRSTS) and Ewing Sarcoma (EWS)	2	Completed	2022-01-21
NCT00001335 (1999-11-03)	New Therapeutic Strategies for Patients with Ewing's Sarcoma Family of Tumors, High Risk Rhabdomyosarcoma, and Neuroblastoma	2	Completed	2002-01
NCT01055314 (2010-01-22)	Temozolomide, Cixutumumab, and Combination Chemotherapy in Treating Patients with Metastatic Rhabdomyosarcoma	2	Completed	2016-06
NCT05093322 (2021-09-20)	A Study of Surufatinib in Combination with Gemcitabine in Pediatric, Adolescent, and Young Adult Patients with Recurrent or Refractory Solid Tumors	1, 2	Completed	2023-04-25
NCT04095221 (2019-09-17)	A Study of the Drugs Prexasertib, Irinotecan, and Temozolomide in People with Desmoplastic Small Round Cell Tumor and Rhabdomyosarcoma	1, 2	Completed	2025-02-18
NCT02581384 (2015-10-19)	Stereotactic Body Radiotherapy (SBRT) for Pulmonary Metastases in Ewing Sarcoma, Rhabdomyosarcoma, and Wilms Tumors	1, 2	Terminated	2020-08
NCT06932861 (2025-04-10)	Exploratory Study of Personalized mRNA Vaccine in Patients with Refractory Rhabdomyosarcoma	1	Not yet recruiting	*2028-05-01
NCT06865664 (2025-03-07)	FGFR4 Chimeric Antigen Receptor (CAR) T Cells in Children and Young Adults with Recurrent or Refractory Rhabdomyosarcoma	1	Not yet recruiting	*2029-04-01
NCT06816771 (2025-02-03)	Evaluation of the Efficacy and Safety of Pazopanib in Combination with TGI/​CIV for Recurrent or Refractory Rhabdomyosarcoma in Children or Adolescents	2	Recruiting	*2026-12-31
NCT06684327 (2024-11-10)	Multi-cohort, Single-arm Phase II Study of Albumin-paclitaxel, Ifosfamide, and Cisplatin in the Treatment of Rare Advanced Tumors	2	Recruiting	*2027-12-30
NCT06456892 (2024-06-07)	Effectiveness of Pucotenlimab Combined with Standard Chemotherapy Regimen	1, 2	Recruiting	*2026-12-06
NCT06094101 (2023-09-25)	Personalized Vaccination in Fusion+ Sarcoma Patients (PerVision)	1, 2	Recruiting	*2027-09
NCT06023641 (2023-08-27)	Treatment of Newly Diagnosed Rhabdomyosarcoma Using Molecular Risk Stratification and Liposomal Irinotecan Based Therapy in Children with Intermediate and High-Risk Disease	2	Recruiting	*2037-10
NCT05304585 (2022-02-24)	Chemotherapy for the Treatment of Patients with Newly Diagnosed Very Low-Risk and Low Risk Fusion Negative Rhabdomyosarcoma	3	Recruiting	*2030-06-30

*: Estimated study completion date

**Table 2:** Development of small molecule drugs targeting RAS pathway

Small molecule drug	Target	Combined agent	Diseases	Phase#	Status	NCT number (Registration Date)
Tunlametinib	MEK	PD-1 antibody	Thyroid Cancer	2	Not yet recruiting	NCT06970353 (2025-05-06)
		None	Melanoma	2	Completed	NCT05217303 (2022-01-07)
		None	Melanoma	3	Recruiting	NCT06008106 (2023-08-08)
Luvometinib	MEK	None	Melanoma	1	Suspended	NCT03932253 (2019-04-25)
Trametinib	MEK	CDX-3379 (ERBB3 antibody)	Melanoma	1, 2	Terminated	NCT03580382 (2018-07-05)
Belvarafenib	RAF	None	Solid Tumor	1	Completed	NCT03118817 (2017-03-20)
Belvarafenib	RAF	Nivolumab (PD-1 antibody)	Melanoma	1	Active, not recruiting	NCT04835805 (2021-04-06)
Cobimetinib	MAP2K1 /MEK1					
Binimetinib	MEK1/2	Bocodepsin (HDAC inhibitor)	Melanoma	1, 2	Completed	NCT05340621 (2022-02-22)
Binimetinib	MEK1/2	None	Advanced refractory cancers, lymphomas, multiple myeloma	2	Active, not recruiting	NCT04439344 (2020-06-18)
BDTX-4933	BRAF	None	Melanoma, histiocytic neoplasms	1	Recruiting	NCT05786924 (2023-03-06)
Mirdametinib	MEK	None	Multiple myeloma	1, 2	Not yet recruiting	NCT06876142 (2025-03-13)
Sirolimus	mTOR					
Onvansertib	PLK1	FOLFIRI, Bevacizumab	Colorectal cancer	2	Completed	NCT05593328 (2022-10-20)
		FOLFIRI, FOLFOX, Bevacizumab	Colorectal cancer	2	Active, not recruiting	NCT06106308 (2023-10-24)
NST-628	RAF, MEK	None	Melanoma, solid tumor	1	Recruiting	NCT06326411 (2024-03-15)
Imalumab	MIF	5-FU/LV	Colorectal cancer	2	Terminated	NCT02448810 (2015-05-15)
Exarafenib	RAF	Binimetinib	Solid tumor	1	Recruiting	NCT04913285 (2021-05-18)
BI 3011441	MEK	None	Solid Tumor	1	Completed	NCT04742556 (2021-02-03)
Ulixertinib	ERK1/2	None	Solid tumor	*Expanded Access	Available	NCT04566393 (2020-09-17)

*: A way for patients with serious diseases or conditions who cannot participate in a clinical trial to gain access to a medical product that has not been approved by the USA FDA. Data were collected form https://www.clinicaltrials.gov/ accessed on 2025-05-30

**Table 3:** FDA approved drugs targeting CDK4/6

Drug	Combination drugs/therapy	Common Adverse events	Status
Palbociclib	Endocrine therapy, fulvestrant, aromatase inhibitor, letrozole, PI3K inhibitor + taselisib (NCT02389842), inavolisib (NCT04191499)	Neutropenia, leukopenia, fatigue, thrombocytopenia	USA FDA approved
Ribociclib	Fulvestrant, aromatase inhibitor, letrozole, everolimus + exemestane (NCT02732119)	Neutropenia, nausea, thrombocytopenia, QTc prolongation, elevated liver enzymes, creatinine increase	USA FDA approved
Abemaciclib	Fulvestrant, nonsteroidal aromatase inhibitor	Diarrhea, neutropenia, asymptomatic serum creatinine increase	USA FDA approved
Dalpiciclib	Pyrotinib, endocrine therapy, fulvestrant	Neutropenia, leukopenia, diarrhea	China FDA approved

**Table 4:** Ongoing and completed clinical trials applying CDK4/6 inhibitors to cancers except for breast cancer

Type of cancer	Drugs/therapies	Phase#	Status	NCT number (Registration Date)
Chordoma	Palbociclib	2	Completed	NCT03110744 (2017-03-31)
Medulloblastoma	Palbociclib, Ribociclib, Abemaciclib	Early 1	Recruiting	NCT06959979 (2024-07-24)
Non-small cell lung cancer	Trilaciclib	2	Recruiting	NCT06328049 (2024-03-13)
	Osimertinib + Dalpiciclib	2	Recruiting	NCT06363734 (2024-04-09)
	Ribociclib + Ceritinib	1	Completed	NCT02292550 (2014-11-12)
Small cell lung cancer	Abemaciclib	2	Recruiting	NCT04010357 (2019-07-03)
	Trilaciclib + Topotecan	4	Recruiting	NCT05874401 (2023-04-12)
Nasopharyngeal carcinoma	Dalpiciclib + Camrelizumab	2	Unknown status	NCT05724355 (2023-02-02)
Meningioma	Abemaciclib	2	Recruiting	NCT05940493 (2023-07-04)
Liposarcoma	Palbociclib + INCMGA00012	2	Active, not recruiting	NCT04438824 (2020-06-17)
Rheumatoid arthritis	TCK-276	1	Completed	NCT05437419 (2022-06-23)
Ovarian Carcinoma	Dalpiciclib + Letrozole	2	Recruiting	NCT06243185 (2024-01-09)
	Abemaciclib	2	Recruiting	NCT04469764 (2020-07-06)
Glioblastoma	Abemaciclib + Bevacizumab	Early 1	Active, not recruiting	NCT04074785 (2019-08-28)
Prostate cancer	Abemaciclib	2	Completed	NCT01739309 (2012-11-29)
	Docetaxel + Ribociclib	1, 2	Completed	NCT02494921 (2015-07-08)
	Palbociclib	2	Active, not recruiting	NCT02905318 (2016-09-08)
	Abemaciclib	2	Completed	NCT04408924 (2020-05-28)
Solid tumor (except breast cancer (however, triple negative was included), liposarcoma, CRPC, melanoma and teratoma) or hematological malignancy (except mantle cell lymphoma)	Ribociclib	2	Completed	NCT02187783 (2014-07-09)

Data were collected form https://www.clinicaltrials.gov/ accessed on 2025-05-30
